# Guiding protein-ligand docking with different experimental NMR-data

**DOI:** 10.1186/1758-2946-4-S1-P39

**Published:** 2012-05-01

**Authors:** Tim ten Brink, Ionut Onila, Adam Mazur, Oliver Korb, Heiko M Möller, Christian Griesinger, Teresa Carlomagno, Thomas E Exner

**Affiliations:** 1 Department of Chemistry, University Konstanz, 78457 Konstanz, Germany; 2 Department of NMR-Based Structural Biology, MPIbpc, 37077 Göttingen, Germany; 3 Structural and Computational Biology Unit, EMBL, 69117 Heidelberg, Germany; 4 Zukunftskolleg, University Konstanz, 78457 Konstanz, Germany

## 

Today's scoring functions are one of the main reasons that state-of-the-art protein-ligand dockings fail in about 20 % to 40 % of the targets due to the sometimes severe approximations they make. However these approximations are necessary for performance reasons. One possibility to overcome these problems is the inclusion of additional, preferably experimental information in the docking process. Especially ligand-based NMR experiments that are far less demanding than the solution of the whole complex structure are helpful.

Here we present the inclusion of three different types of NMR-data into the ChemPLP [1] scoring function of our docking tool PLANTS [2]. First, STD and intra-ligand trNOE spectra were used to obtain distant constraints between ligand and protein atoms. This approach proved beneficial for the docking of larger peptide ligands i. e. the epitope of MUC-1 glycoprotein to the SM3 antibody [3].

In the second part the usefulness of INPHARMA data [4,5] is shown by combinig a score, evaluating the agreement between simulated and measured INPHARMA spectra, with the PLANTS ChemPLP scoring function. First results from rescoring after local optimization of the poses and full docking experiments are shown.
